# Moiré-Induced Electronic Reconstruction in van der Waals Heterobilayer PtSe_2_/PtTe_2_

**DOI:** 10.1021/acsnano.5c19273

**Published:** 2026-02-02

**Authors:** Yin-Song Liao, Ruei-Yu Wang, Han-Wei Tsai, Guan-Hao Chen, Hsin-Hsien Chan, Hsun-Ting Hsieh, Cheng-Maw Cheng, Chun-Liang Lin, Meng-Kai Lin, Jyh-Pin Chou

**Affiliations:** † Graduate School of Advanced Technology, 33561 National Taiwan University, Taipei 106319, Taiwan; ‡ Department of Physics, 34911 National Central University, Taoyuan 32001, Taiwan; § Department of Electrophysics, 34914 National Yang Ming Chiao Tung University, Hsinchu 300039, Taiwan; ∥ 57815 National Synchrotron Radiation Research Center, Hsinchu 30076, Taiwan

**Keywords:** moiré superlattice, van der Waals heterostructures, interfacial hybridization, ARPES, flat bands, stacking registry, PtSe_2_/PtTe_2_

## Abstract

van der Waals heterostructures composed of a few atomic layers have attracted significant attention in the condensed matter physics community. Although interlayer bonding is weak, effects such as moiré modulation, charge redistribution, and electronic hybridization can substantially modify the band structure and interlayer coupling. In this work, we investigate heterostructures composed of few-layer PtSe_2_ and PtTe_2_ by using first-principles calculations implemented within density functional theory (DFT) and angle-resolved photoemission spectroscopy (ARPES). While both materials are Dirac semimetals in the bulk form, they undergo a transition to semiconducting states in the few-layer limit. These heterostructures allow systematic examination of how dimensional confinement and interfacial interactions influence band structure and interlayer coupling. Our combined ARPES measurements and DFT calculations indicate the presence of electronic hybridization at the interface. The interlayer coupling in PtSe_2_/PtTe_2_ is associated with flat-band features and valence-band splitting induced by both inversion symmetry breaking and spin–orbit coupling. Furthermore, the local density of states indicates metallic behavior at the MM site while it remains semiconducting at MX and XX sites with the band gap of 0.40 and 0.25 eV, respectively. Further analysis shows that the electronic hybridization and charge transfer between PtSe_2_ and PtTe_2_ are sensitive to the interlayer distance, which is consistent with moiré characteristics. These results highlight how interfacial interactions govern the electronic properties of vdW heterostructures.

## Graphical abstract

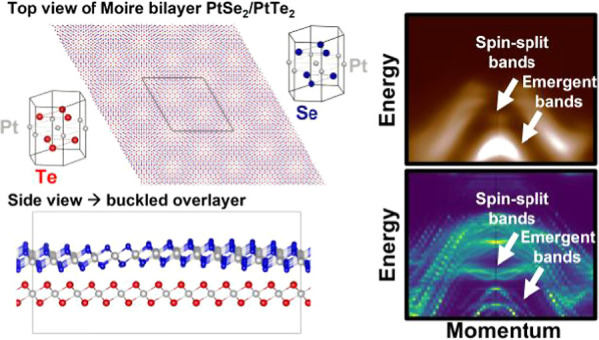

## Introduction

Two-dimensional layered materials, particularly transition-metal dichalcogenides (TMDs), have attracted significant attention in recent years owing to their successful synthesis down to the few-atomic layer or even the monolayer limit. TMDs are composed of one transition metal and two chalcogens, forming a family with stoichiometry of MX_2_ (M is the transition metal and X is the chalcogen atom) encompassing widely studied members such as the MoS_2_, WSe_2_, PtSe_2_, and PtTe_2_.^1–4^ Their electronic properties exhibit a strong thickness dependence: for example, semiconducting MoS_2_ and WSe_2_ transitions from an indirect to a direct band gap as their structure is reduced from bulk to single layer.^5–7^ Such tunability, together with their mechanical flexibility and chemical stability, makes TMDs promising candidates for next-generation nanoelectronics, optoelectronics, and catalytic applications, including field-effect transistors, photodetectors, and electrocatalysts.^8–12^ Generally, TMD layers are covalently bonded in the lateral direction but are held together by relatively weak van der Waals (vdW) forces in the out-of-plane direction. This characteristic provides the playground by stacking different TMD layers to form vdW heterostructure^13,14^ with atomically sharp interfaces. Although vdW forces are relatively weak compared to those of covalent bonds, the interlayer coupling could be remarkably strong, often giving rise to emergent phenomena not present in the constituent monolayers. For instance, the TiTe_2_/TiSe_2_ heterostructures exhibit moiré-induced band modulation and coherent interlayer hybridization, leading to band dispersions that deviate from those of the individual layers.^15^ Similarly, PtTe/PtTe_2_ heterostructures display giant Rashba-type spin splitting and symmetry-breaking-induced electronic states, as revealed by second harmonic generation measurements and spin-resolved angle-resolved photoemission spectroscopy (ARPES).^16^

The electronic structure of vdW heterostructures is further influenced by the local stacking registry and the associated interlayer spacing. Previous studies on bilayer PtSe_2_ and bilayer PtTe_2_ have shown that the equilibrium interlayer distance varies with the stacking configuration (AA, AB, or AB′), which is likely governed by the interaction between chalcogen atoms at the interface.^17–19^ Such sensitivity to the local atomic registry suggests that even subtle geometric variations can lead to noticeable structural changes in both structural corrugation and electronic coupling. The interplay between structural reconstruction and electronic modulation is a hallmark of moiré heterostructures. Several theoretical studies on moiré heterostructures composed of structurally similar TMDs have demonstrated that stacking-dependent interlayer coupling and local structural reconstruction can lead to pronounced electronic modulation. A representative example is the MoS_2_/MoSe_2_ bilayer, in which moiré-induced variations in local stacking and interlayer distance give rise to wave function localization and band-edge modulation.^20^ More broadly, moiré superlattices provide a versatile platform for hosting emergent phenomena such as moiré excitons,^21,22^ gate-tunable Mott insulators,^23^ and novel functionalities in photonics and optoelectronics systems.^24–26^ While these studies have established key principles of moiré engineering in semiconducting 2H-phase TMDs, comparatively fewer studies have explored similar-material moiré heterostructures in systems with strong spin–orbit coupling (SOC) and different crystal symmetries. Understanding how subtle interlayer hybridization, symmetry breaking, and SOC-driven band reconstruction manifest in such systems therefore remains an important open question, which motivates the present study of vdW heterostructures.

Recently, Li et al. reported an electronically textured PtSe_2_/PtTe_2_ using scanning tunneling microscope/spectroscopy (STM/STS) combined with density functional theory (DFT) calculation,^27^ revealing an electronically corrugated moiré landscape. The authors attributed the observed STM contrast mainly to electronic effects, inferring minimal topographic buckling. However, the absence of direct evidence for geometric modulation raises an open question regarding how stacking-dependent structural corrugation contributes to the electronic properties of PtSe_2_/PtTe_2_.

Here, we present a combined theoretical-experimental investigation of PtSe_2_/PtTe_2_ heterostructures across a wide range of stacking configurations and thickness combinations. Our first-principles DFT calculations reveal strong interlayer coupling, pronounced geometric corrugation, and SOC-induced band modifications, with the interlayer distance varying locally depending on the stacking registry. We employed molecular beam epitaxy (MBE) to grow high-quality, well-oriented PtSe_2_/PtTe_2_ heterostructures. Owing to the large nearly single-domain regions achieved by MBE growth, our ARPES measurements were able to resolve the high-symmetry directions in momentum space. Our ARPES results directly confirm the emergent band dispersions anticipated by DFT calculations and highlight the critical role of geometric buckling and SOC in modulating the electronic structure. Furthermore, we demonstrate that the band characteristics can be tuned by controlling the thickness of the PtSe_2_ and PtTe_2_ layers. To the best of our knowledge, this is the first integrated theoretical-experimental study that systematically elucidates both the geometric and the electronic structures of PtSe_2_/PtTe_2_ heterostructures.

## Results and Discussion

The electronic structure of PtTe_2_ and PtSe_2_ exhibits a pronounced dependence on layer thickness.^4,28^ Our DFT calculations show that monolayer PtTe_2_ (PtSe_2_) is semiconducting with a band gap of 0.28 eV (1.15 eV). For PtSe_2_, the gap decreases rapidly as the number of layer increases, from 0.34 eV in 2-trilayer (2TL) to 0.11 eV in 3TL, whereas PtTe_2_ becomes metallic beyond the monolayer limit. The corresponding total- and orbital-projected band structures for different thicknesses are provided in Figures S5 and S6. This layer-dependent transition establishes the foundation for understanding the hybridized electronic states in the PtTe_2_/PtSe_2_ heterostructure.

Figure 1a compares the ARPES maps with the calculated DFT band structures of monolayers PtSe_2_ (blue) and PtTe_2_ (red). The corresponding second-derivative maps in Figure 1b provide enhanced visualization of the band dispersions, showing excellent agreement between the experiment and theory for the pristine layers. If the PtSe_2_/PtTe_2_ interface behaved as an electronically inert vdW contact, its bands would represent a simple superposition of the two monolayers. Instead, pronounced deviations appear near the Γ-point, where the valence band dispersions merge into distinct eye-shaped bands centered at around *E* = −2.1 eV. This emergent feature, absent in either constituent, reveals the formation of hybridized interfacial states driven by strong interlayer coupling.

**Figure 1:**
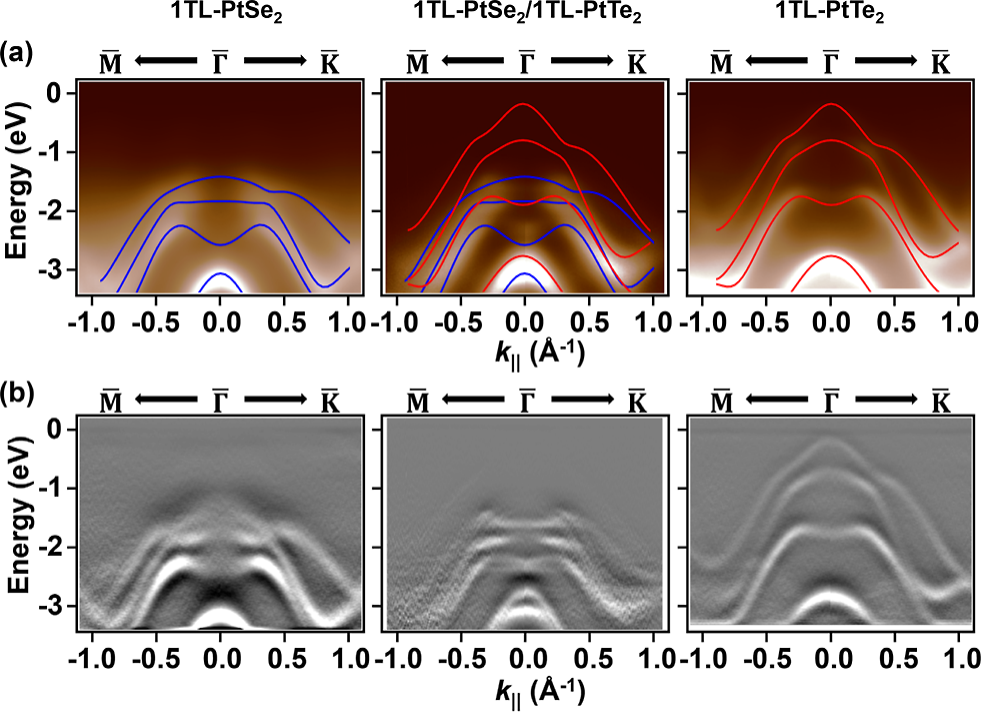
(a) ARPES maps and corresponding DFT band structures of 1TL-PtSe_2_, 1TL-PtSe_2_/1TL-PtSe_2_, and 1TL-PtTe_2_ along the M–Γ–K *k*-path. The calculated dispersions of 1TL-PtSe_2_ and 1TL-PtTe_2_ are superimposed as blue and red lines, respectively. (b) Second-derivative ARPES maps of the same three systems, highlighting the dispersive features.

To elucidate the microscopic origin of these states, we performed comprehensive DFT calculations on a commensurate heterostructure composed of a 1TL-PtSe_2_ (13 × 13) supercell stacked on a 1TL-PtTe_2_ (12 × 12) supercell without rotation (denoted as 0°-model, see Figures 2a and S7). Experimentally, the heterostructure was grown by MBE on a bilayer-graphene-terminated SiC(0001) (BLG for short), which acts as an electronically inert substrate owing to the weak interaction between its delocalized π electrons and TMD layers.^29^ Hence, the substrate effect is neglected in our simulation. To mimic this steric constraint from the substrate, the *z*-coordinates of the bottom Te atoms in PtTe_2_ were fixed during structural relaxation, as indicated by the green-shaded region in Figure 2b, and all other atoms were fully optimized. Several lattice parameters were also examined, and their relative stability comparisons are present in Figure S7.

**Figure 2:**
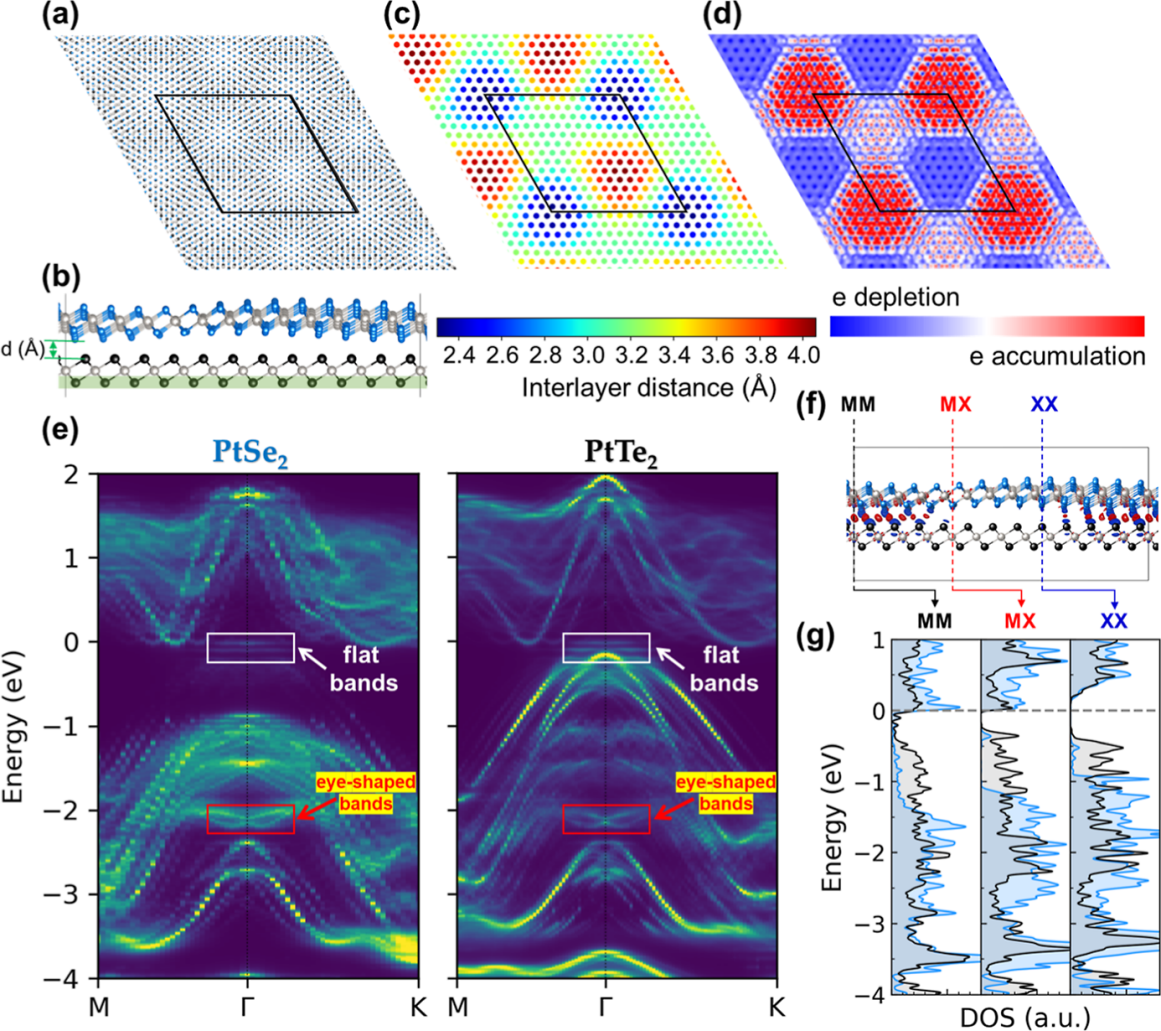
(a) Top view of the 1TL-PtSe_2_/1TL-PtTe_2_ heterostructure. (b) Side view of the same heterostructure; Pt, Se, and Te atoms are shown in silver, blue, and black spheres, respectively. (c) Spatial profile of the interlayer distance of the commensurate PtSe_2_ (13 × 13) and PtTe_2_ (12 × 12). (d) Charge density difference contour plot in the interfacial region between PtSe_2_ and PtTe_2_. The black lines denote the cell boundaries. (e) Layer-projected band structure of the 1TL-PtSe_2_/1TL-PtTe_2_ heterostructure. The energy is aligned to the Fermi level. (f) Side view of the charge density difference; the red and blue lobes represent charge accumulation and depletion region, respectively. (g) Local density of states at representative MM, XX, and MX stacking sites; the blue and black curves correspond to the PtSe_2_ and PtTe_2_ contributions, respectively.

Upon structural optimization, the PtTe_2_ layer retains its nearly pristine geometry with negligible lattice distortion, whereas the PtSe_2_ develops pronounced buckling that persists under a different strain-distribution scheme (see Figure S7). This corrugation gives rise to spatially varying interlayer spacing across the PtSe_2_/PtTe_2_ interface, as illustrated in Figure 2c. The nonuniform spacing originates from a moiré-type potential arising from local stacking variations. Consistent with previous reports bilayer PtSe_2_ and bilayer PtTe_2_,^17,18^ the interlayer distance is highly sensitive to stacking configuration, displaying distinct separations for AA, AB, and AB′ registries. The heterostructure follows the same trend, with stacking-dependent interlayer separations governed by the relative alignment of the chalcogen sublayers. To probe the impact of this structural modulation on charge redistribution, we calculated the charge density difference contours presented in the top-view in Figure 2d and side-view in Figure 2f. The resulting patterns exhibit a clear moiré-like modulation that mirrors the spatial variation of the interlayer spacing: regions with larger separations show reduced electron accumulation, while smaller separations exhibit enhanced charge transfer. These results demonstrate that interfacial PtSe_2_/PtTe_2_ is highly localized and governed jointly by both a stacking registry and interlayer spacing.

The layer-projected band structures presented in Figure 2e capture the characteristic eye-shaped band splitting near *E* = −2.1 eV consistent with the APRES spectra in Figure 1b, and interestingly, there are few nearly flat bands close to the Fermi level. Projection onto individual layers reveals that these features arise from the hybridization between PtSe_2_ and PtTe_2_ as the eye-shaped band feature was mainly contributed from PtSe_2_ and the flat band feature was mainly contributed from PtTe_2_. Our calculations predict the presence of flat bands near the Fermi level, which, however, are not observed in the ARPES spectra. This discrepancy is likely due to the limited probing depth of photoelectrons as these flat bands mainly originate from the PtTe_2_ substrate. Although pristine monolayers PtSe_2_ and PtTe_2_ are semiconducting, their direct contact renders the 1TL-PtSe_2_/1TL-PtTe_2_ stack metallic, reflecting strong interlayer coupling. This metallic feature can be experimentally observed, with a faint but highly dispersed band across the Fermi level in the second-derivative ARPES map (Figure 1b). Beyond the eye-shaped band splitting, an extra set of nearly flat bands around the Fermi level indicates the emergence of hybridized interfacial states unique to the heterostructure. Comparison with the band structure calculated without SOC effects (Figure S8) shows that the eye-shape splitting feature vanishes in the absence of SOC, indicating that SOC plays a critical role in generating these distinctive electronic features. Such momentum-dependent band splitting is characteristic of the Rashba effect, which arises from SOC in systems lacking inversion symmetry and leads to spin-momentum locking in the electronic states. In this context, the eye-shaped splitting provides a direct and experimentally relevant fingerprint of inversion-symmetry breaking and SOC-driven band reconstruction induced by the PtSe_2_/PtTe_2_ heterointerface.

The spatial charge density difference in Figure 2f further reveals that charge transfer in PtSe_2_/PtTe_2_ varies with local stacking. Three representative registries, i.e., MM, XX, and MX, are identified according to the relative positions of metal (M) and chalcogen (X) sublattices across the interface. The corresponding local density of states (LDOS) at these sites is shown in Figure 2g, revealing metallic behavior at the MM site and semiconducting character at the MX and XX sites. The most pronounced electron accumulation occurs at the MM site, arising from strong Se–Te hybridization at the interface, which drives the LDOS there into a metallic state. In contrast, the MX and XX sites demonstrate finite band gaps of 0.4 and 0.25 eV, respectively. For reference, the band gaps obtained from our DFT calculations for the pristine 1TL-PtSe_2_, 2TL-PtSe_2_, and 1TL-PtTe_2_ are 1.32, 0.37, and 0.66 eV, respectively.

To isolate the contribution of interfacial coupling from that of geometric corrugation, we recalculated the band structure of 1TL-PtSe_2_/1TL-PtTe_2_ using optimized interlayer distances of 3.4 Å while fixing all atomic positions to preserve the intrinsic monolayer geometry. As shown in Figure S9, the eye-shaped splitting bands near *E* = −2.1 eV remain, whereas the flat band feature vanishes. This confirms that the flat bands are closely linked to geometric buckling, while the eye-shaped dispersion arises primarily from electronic hybridization.

If the direct contact between semiconducting PtSe_2_ and PtTe_2_ could result in the strong interfacial hybridization and even lead to the emergence of the additional band features such as eye-shaped splitting bands and exotic flat bands, it is wondered how the interaction behavior deviates with the different number of PtTe_2_ substrate layers as the metallicity of PtTe_2_ increases with the number of layers. To examine how the degree of interfacial coupling evolves with the increasing metallicity of PtTe_2_, we investigated the thickness-dependent band structure of 1TL-PtSe_2_/*m*TL-PtTe_2_ (*m* = 1–3). It is noted that the computational cost of the large PtSe_2_ (13 × 13) on the PtTe_2_ (12 × 12) supercell model is extremely expensive, which is challenging for the multilayer PtTe_2_ configurations. We adopted a reduced and rotated model, i.e., 1TL-PtSe_2_ (√84 × 1) stacked on *m*TL-PtTe_2_ (√73 × 1) with a rotation angle of ∼5°, denoted as 5°-model. The SOC effect could be included in all calculations by this smaller model. The detailed difference between two models can be referred to the computational details. As noted previously, PtSe_2_ undergoes obvious lattice distortion upon heterostructure formation. To disentangle the influence of such geometric distortion effects on the electronic structure, we calculated individual band structures of the distorted but freestanding monolayers extracted from the as-optimized heterostructure, as shown in Figure S10. Though PtTe_2_ experiences only negligible lattice distortion, PtSe_2_ exhibits drastic buckling after structural optimization, which is consistent with the 0°-model. Nevertheless, the electronic structure of the distorted freestanding layers remain nearly identical to those of primitive counterparts, but only energy bands become more dispersive spreading in momentum space. This illustrates the strain arising from the lattice mismatch, and the buckling in PtSe_2_ and PtTe_2_ lattice barely influences the electronic structures of freestanding layers.

In the 0°-model, the characteristic eye-shaped band centered at *E* = −2.1 eV, the red box in Figure 2e, reappears in the 5°-model, as indicated by the white arrow in Figure 3b. As the substrate PtTe_2_ increases to 2TL and 3TL, the APRES maps and their second derivative counterparts, see Figure 3c,d, show that interlayer coupling between the top PtSe_2_ and the underlying PtTe_2_ persists, although the eye-shaped feature gradually weakens. Our DFT results reveal a corresponding reduction in the PtTe_2_ contribution, consistent with the diminishing hybridization strength. Additional spectral features marked by red arrows in Figure 3b originate from orbital coupling between the d_*z*_^2^ orbital of PtSe_2_ and PtTe_2_ (Figure S11), while the eye-shaped splitting stems from the hybridization of p_*z*_ orbitals of Se and Te. Together, the ARPES and DFT results confirm that strong electronic hybridization occurs across the interface regardless of the PtTe_2_ thickness. The eye-shaped dispersion arises from the interplay of interlayer hybridization, SOC, and geometric buckling. Hybridization introduces new interfacial states, and SOC further lifts their degeneracy through inversion-symmetry breaking at the corrugated interface, producing the characteristic eye-shaped subbands.

**Figure 3:**
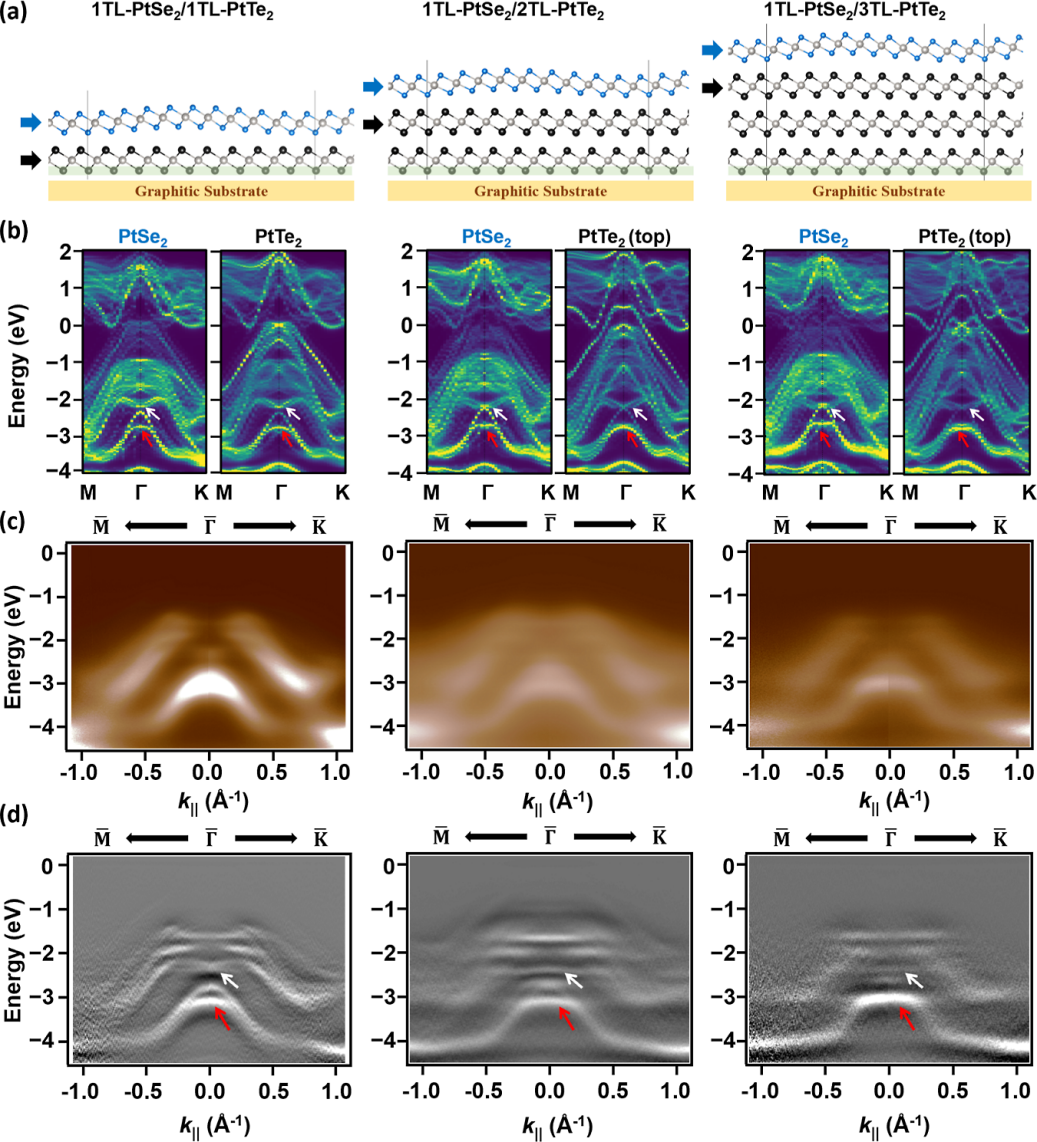
(a) Geometries of 1TL-PtSe_2_/*m*TL-PtTe_2_ (*m* = 1–3) heterostructure. The *z*-coordinates of Te atoms in the green-shaded area are fixed to emulate the substrate constraint; all other atoms are allowed to relax. The white and red arrows highlight the two key features discussed in the text. (b) Corresponding layer-projected band structures; blue and black arrows indicate contributions from PtSe_2_ and PtTe_2_, respectively. (c) ARPES spectra along the M–Γ–K *k*-path for 1TL-PtSe_2_/*m*TL-PtTe_2_ (*m* = 1–3). (d) Corresponding second-derivative maps in (c).

To visualize the spatial nature of these states, we analyzed the partial charge densities in three representative energy windows, labeled A–C in Figure 4a. Region A corresponds to the flat bands near the Fermi level, whose charge density exhibits a pronounced *C*_3_-symmetry and strong localization at the MM site. As shown in Figure S12, this strong localization persists across different isovalues, indicating that the observed confinement is intrinsic to the electronic structure and independent of the visualization parameters. The plane-averaged charge density along the *z*-direction shows a sharp peak at the interface (purple-shaded region), indicating pronounced interlayer hybridization due to localized wave function overlap. Region B reveals a moiré-like modulation consistent with the superlattice periodicity. Region C represents the eye-shaped band splitting characterized by spatially delocalized charge distribution across the interface. These observations collectively demonstrate that the flat bands are associated with localized interlayer states, while the eye-shaped dispersions arise from delocalized hybridized orbitals of PtSe_2_ and PtTe_2_.

**Figure 4:**
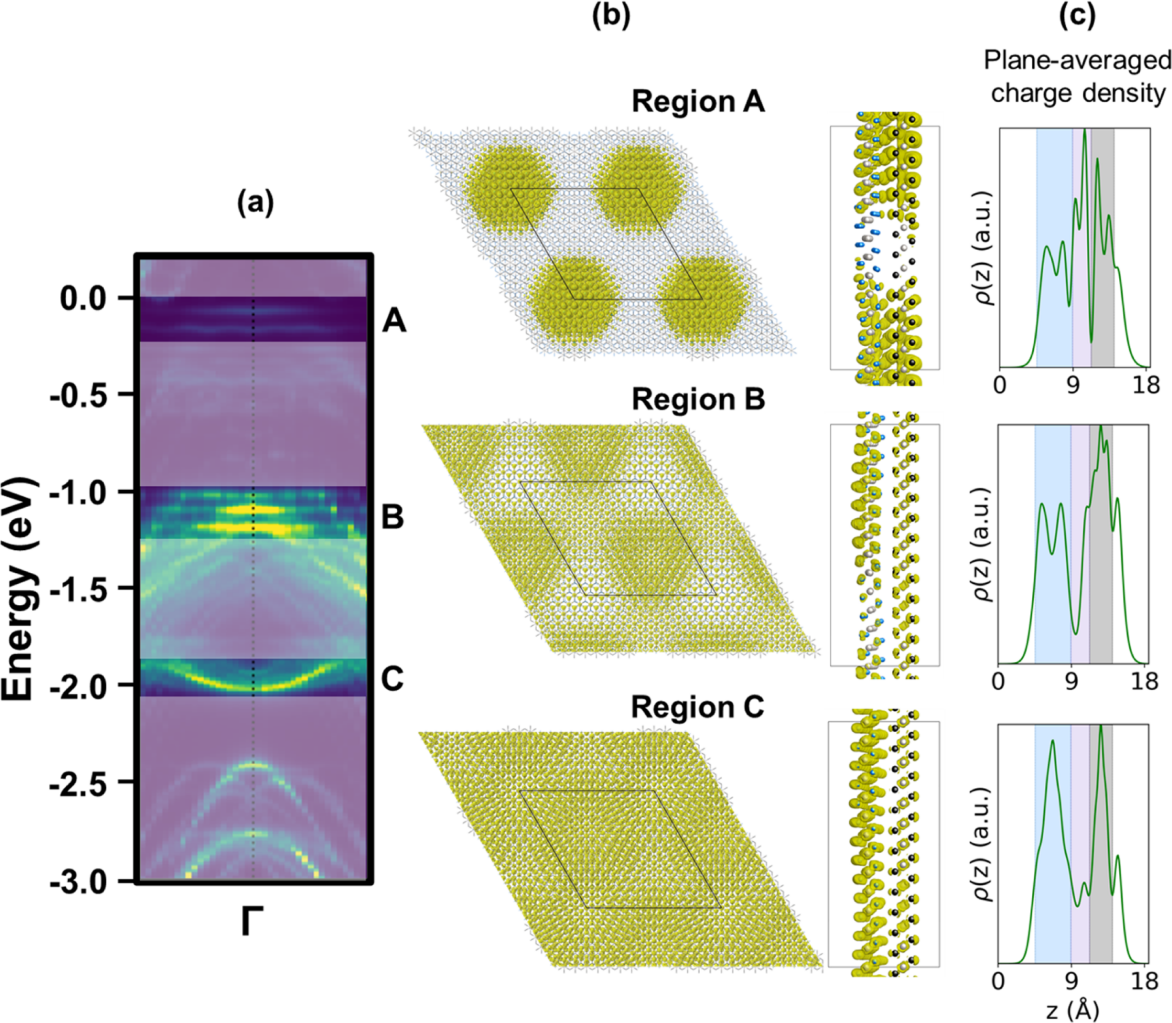
(a) Projected band structure of PtSe_2_ in the PtSe_2_/PtTe_2_ heterostructure model without SOC effect. For the sake of clarity, the partial charge densities at Γ point are highlighted for three distinct energy windows, labeled as A, B, and C, and the rest parts are shadowed. (b) Corresponding 3D partial charge density isosurfaces in top and side views. (c) Plane-averaged partial charge densities along the *z*-direction, where the regions of PtSe_2_, PtTe_2_, and interface are highlighted within blue, black, and purple shades, respectively.

## Conclusion

In conclusion, we have demonstrated that the PtSe_2_/PtTe_2_ heterostructure exhibits pronounced interlayer couplings, giving rise to emergent band features such as flat bands and eye-shaped splitting bands in the valence-band region, as evidenced by both first-principles DFT calculations and ARPES measurements. Our DFT results reveal that geometric buckling strongly modulates the electronic structure, while the excellent agreement between ARPES spectra with the calculated bands further validates the reliability of our theoretical framework. The analysis of interlayer distance, charge density difference, LDOS, and partial charge density highlights the moiré-induced characteristics of the heterostructure. The interlayer spacing between PtSe_2_ and PtTe_2_ exhibits anisotropy arising from the stacking-dependent moiré potential at the interface. The charge density difference further uncovers nonuniform charge redistribution across the interface, leading to spatial variations in hybridization strength within the moiré unit cell. LDOS at the MM site reveals metallic character due to strong Se–Te wave function overlap, whereas the MX and XX sites display semiconducting characteristic with band gaps of 0.40 and 0.25 eV, respectively. Moreover, the partial charge density underscores the localized nature of the flat bands near the Fermi level, while the emergent band around −2.1 eV manifests a distinctly delocalized character. Collectively, these findings establish the PtSe_2_/PtTe_2_ heterostructure as a prototypical platform for exploring interfacial coupling and moiré-driven electronic phenomena in 2D vdW materials.

## Methodology

### Computational Details

The first-principles DFT calculation was implemented within the Vienna Ab initio Simulation Package (VASP) within the plane-wave projector augmented-wave method.^30,31^ The exchange-correlation functional was employed within generalized gradient approximation with the scheme of Perdew–Burke–Ernzerhof (PBE).^32^ The well-converged cutoff energy of 400 eV was used for the plane-wave expansion, and the *k*-points of 12 × 12 × 9 were used to sample the Brillouin zone of bulk PtSe_2_ and bulk PtTe_2_. With a view to taking the vdW dispersion interactions, the revised B86b vdW density functional (B86R) was included in all the calculation.^33^ The SOC effect is included in all band structure calculations unless it is specified excluded. Band unfolding was conducted through the *easyunfold* package.^34^ The in-plane lattice constant of the primitive PtSe_2_ and PtTe_2_ is 3.72 Å and 3.98 Å, respectively, which are consistent with the literature reports.^35,36^

To minimize the strain in the heterostructure caused by lattice mismatch, we initially considered a supercell configuration of PtSe_2_ (13 × 13) and PtTe_2_ (12 × 12), which results in a mismatch of 1.2%. We examined several lattice parameter choices and relaxation protocols, denoted as the “Te-fixed” and “Se-fixed” configurations (see Supporting Information for details). In the primary calculations reported here, the heterostructure was constrained to the mean in-plane lattice constant of the two constituents (∼3.85 Å), resulting in a compressive strain of 0.6% in PtSe_2_ and a corresponding tensile strain of 0.6% in PtTe_2_. This supercell contains 939 atoms, rendering multilayer heterostructure calculations computationally prohibitive. Therefore, to reduce the computational cost while retaining the essential interlayer physics, we constructed an alternative commensurate heterostructure using the *Hetbuilder* package,^37^ which is designed for generating commensurate twisted bilayer geometries. A relative rotation angle of ∼5° was introduced, realized by stacking monolayer PtSe_2_ (√84 × 1) on monolayer PtTe_2_ (√73 × 1) while using the average lattice constant. The reduced model contains only 57 atoms, resulting in a minimum residual strain within the heterostructure. Subsequent calculations revealed that such a small residual strain has a negligible effect on the electronic structure, with the interlayer coupling dominating the evolution of the band dispersion in the heterostructure. A vacuum thickness larger than 10 Å was employed to eliminate the fictitious interaction caused by the period boundary conditions. Experimentally, the rotation angles within 5° were found through the low-energy electron diffraction (LEED) (see Figure S1).

## Experiments

Bilayer-graphene-terminated 6H-SiC(0001) substrates were prepared by repeated flash-annealing cycles. PtTe_2_ films were subsequently grown on these substrates at a deposition rate of approximately one layer per hour, by codepositing Pt and Te with a flux ratio of ∼1:50, while maintaining the BLG substrates at 190 °C. Monolayer PtSe_2_ on PtTe_2_ films was prepared similarly, except that Se was used as the evaporation source instead of Te. The RHEED patterns indicate the high quality of all samples grown with a single domain (see Figure S2). The electronic band structures were characterized by ARPES using 50 eV photons and a Scienta DA30 analyzer at TPS Beamline 39A, National Synchrotron Radiation Research Center (NSRRC). All ARPES measurements were conducted at 40 K. The coverage of PtTe_2_ and PtSe_2_ film growths is based on a comparison of our ARPES and DFT calculations (see Figures S3–S6).

## Supplementary Material



## Notes

LEED and RHEED characterization of PtSe_2_/PtTe_2_ heterostructures, thickness-dependent ARPES spectra of PtSe_2_ and PtTe_2_ films, calculated band structures of pristine PtSe_2_ and PtTe_2_, details of strain-distribution schemes and structural relaxation protocols for commensurate heterostructure models, total-energy comparisons of different lattice constraints, unfolded band structures with and without SOC, analysis of interlayer-distance-dependent electronic structures, orbital-projected band structures, and robustness tests of partial charge density localization (PDF)
