# Supplementary material for: Moiré-Induced Electronic Reconstruction in van der Waals Heterobilayer PtSe2/PtTe2
Source: ACS Nano. 2026 Feb 2;20(6):5102–9. doi: 10.1021/acsnano.5c19273 (PMC12918719; doi:10.1021/acsnano.5c19273)
Supplement: Supplementary file 1 [file nn5c19273_si_001.pdf]

Moiré-Induced Electronic Reconstruction in van der Waals Heterobilayer $PtSe_2/PtTe_2$

Yin-Song Liao[1,†], Ruei-Yu Wang[2,†], Han-Wei Tsai[2,†], Guan-Hao Chen[3], Hsin-Hsien Chan[2], Hsun-Ting Hsieh[2], Cheng-Maw Cheng[4], Chun-Liang Lin[3], Meng-Kai Lin[2,*], and Jyh-Pin Chou[1,*]

1. Graduate School of Advanced Technology, National Taiwan University, Taipei 106319, Taiwan.
2. Department of Physics, National Central University, Taoyuan 32001, Taiwan.
3. Department of Electrophysics, National Yang Ming Chiao Tung University, Hsinchu 300039, Taiwan.
4. National Synchrotron Radiation Research Center, Hsinchu 30076, Taiwan.

† Equal contribution
* Corresponding authors: mklin@office365.ncu.edu.tw; jpchou@ntu.edu.tw

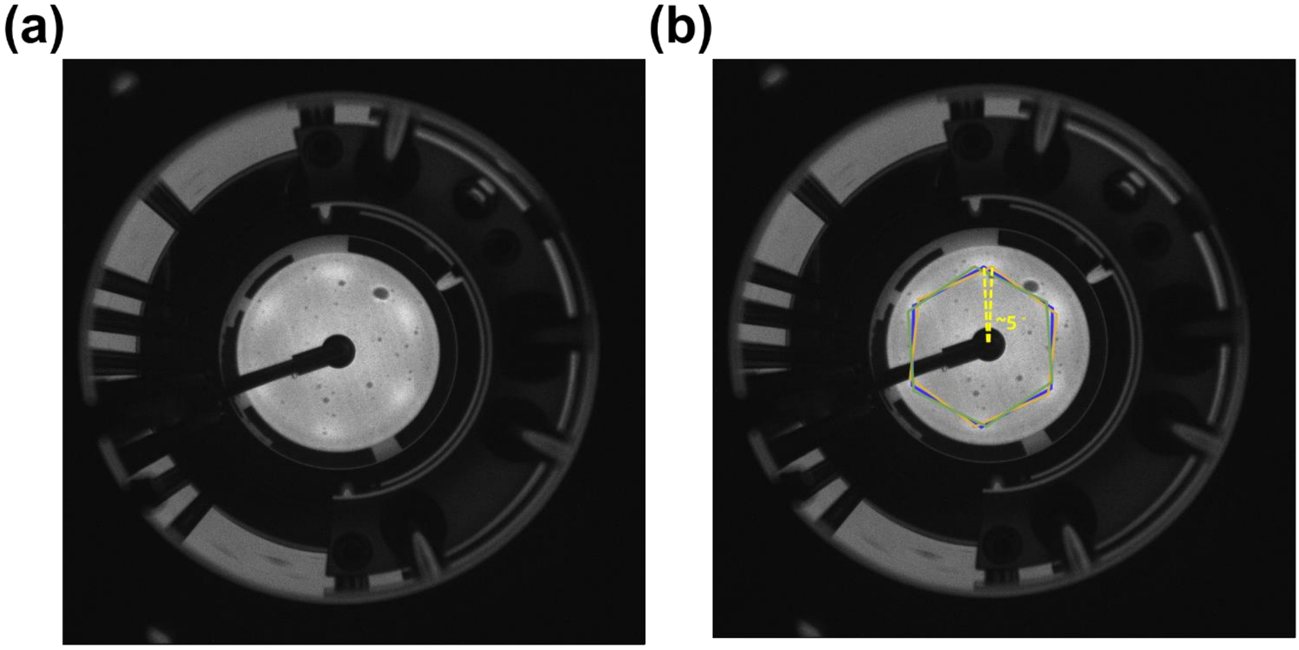

Figure S1. (a) LEED pattern of 1TL-$PtSe_2$/1TL-$PtTe_2$. (b) Same as (a), but with the (1 × 1) surface Brillouin zones attached.

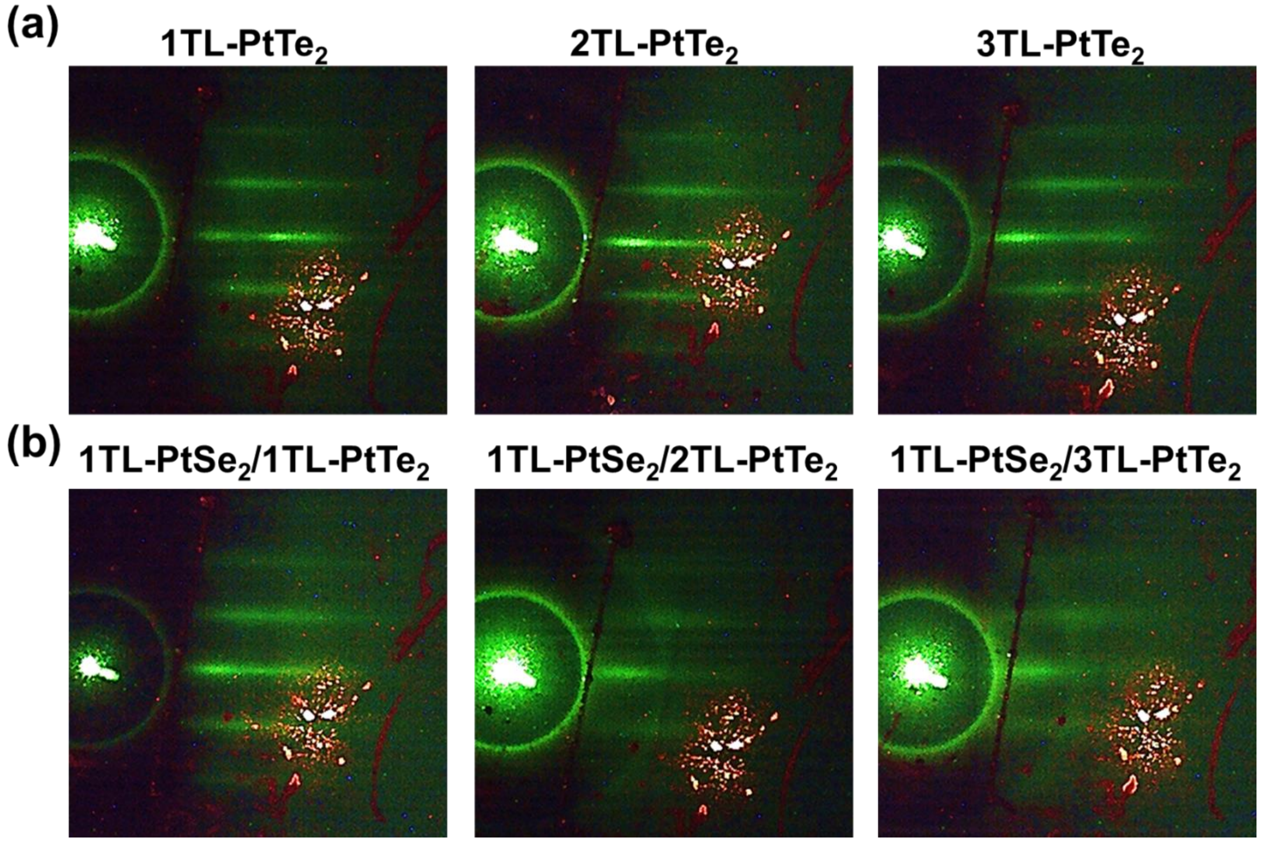

Figure S2. RHEED patterns of (a) $PtTe_2$ of of 1TL-, 2TL-, and 3TL, respectively. (b) 1TL-$PtSe_2$ grown on 1TL-, 2TL-, and 3TL-$PtTe_2$, respectively. The RHEED patterns indicate the high quality of all samples are grown with single domain.

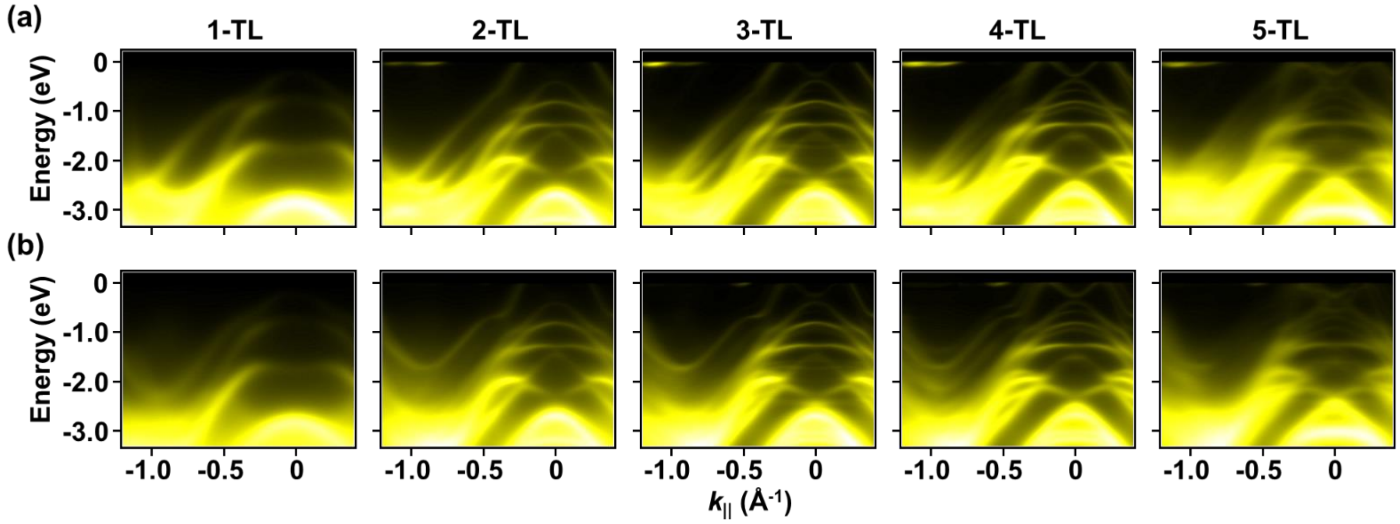

Figure S3. (a) ARPES maps along the ΓK direction taken from 1TL to 5TL of $PtTe_2$ at 20 K using 50 eV photons. (b) Similar to (a), but along the ΓM direction.

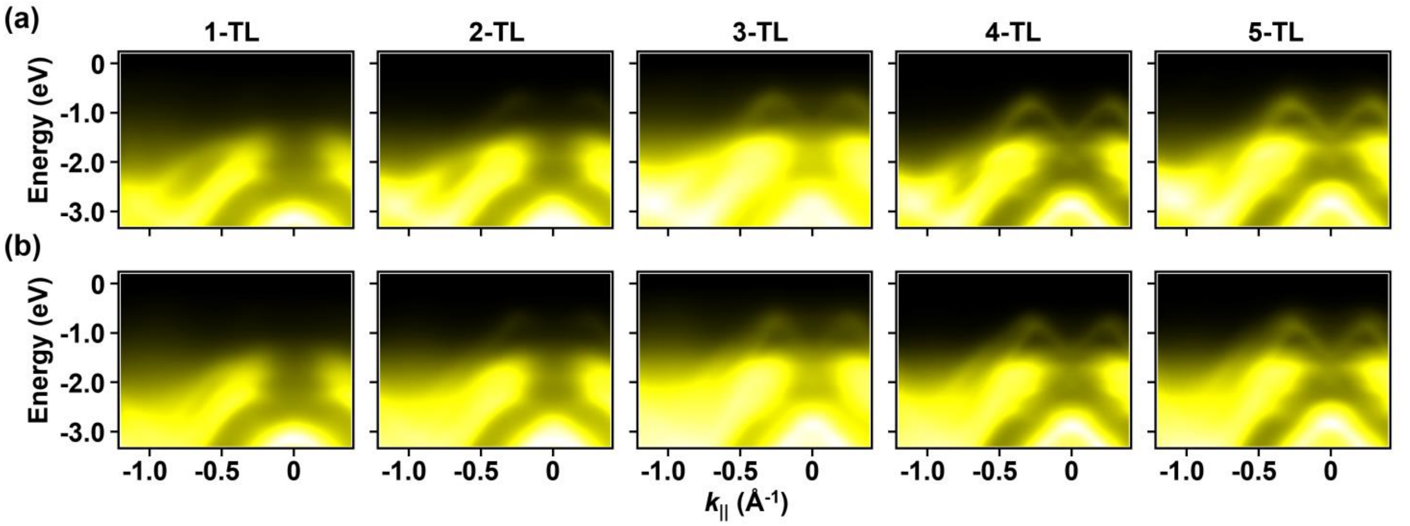

Figure S4. (a) ARPES maps along the ΓK direction taken from 1TL- to 5TL- of $PtSe_2$ at 20 K using 50 eV photons. (b) Similar to (a), but along the ΓM direction.

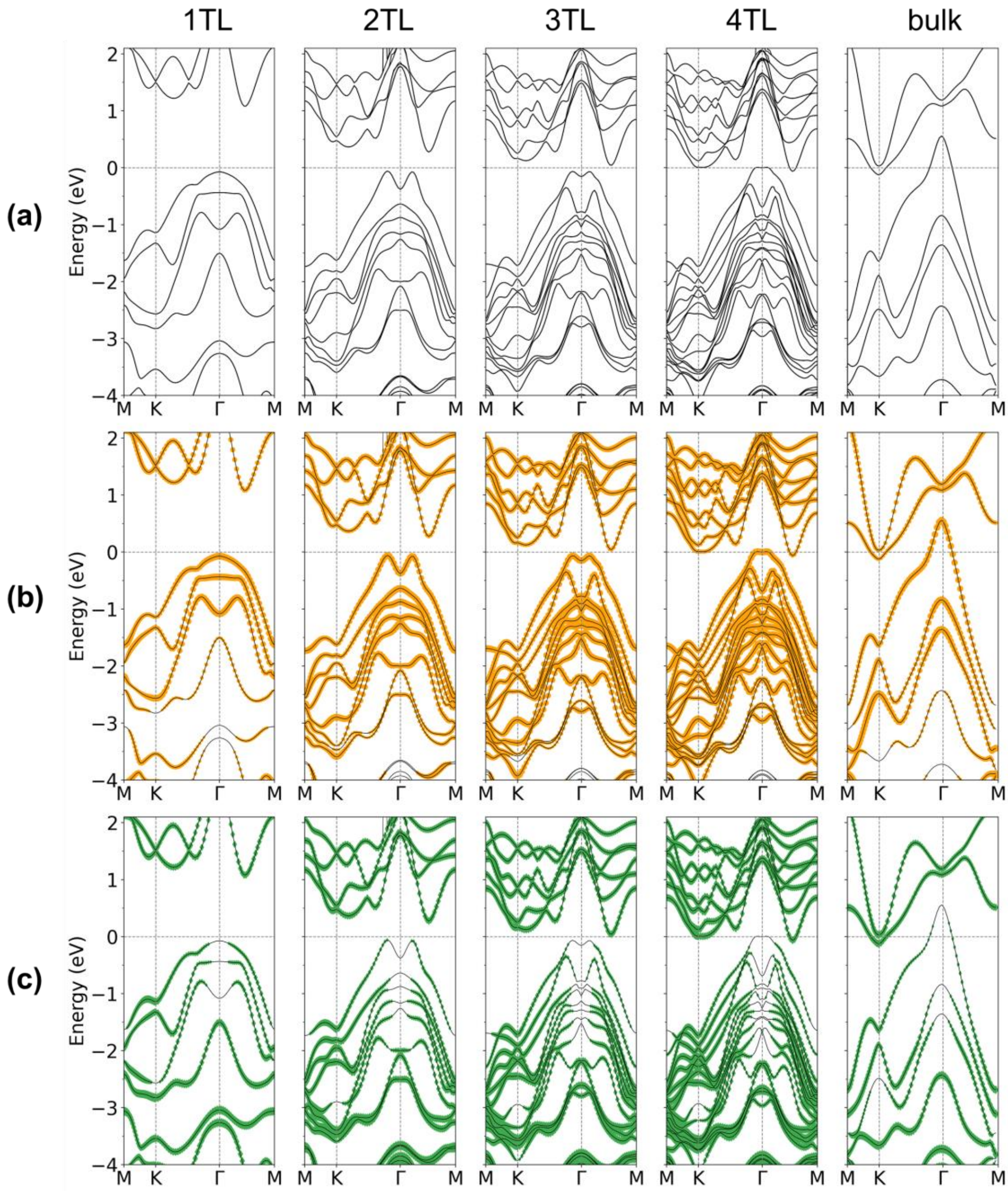

Figure S5. Calculated band structure of primitive $PtSe_2$. (a) Total band structure; Projected band structure for (b) p-orbital (orange spheres) and (c) d-orbital (green diamond), where the size of the symbol indicates the orbital contribution at each k point.

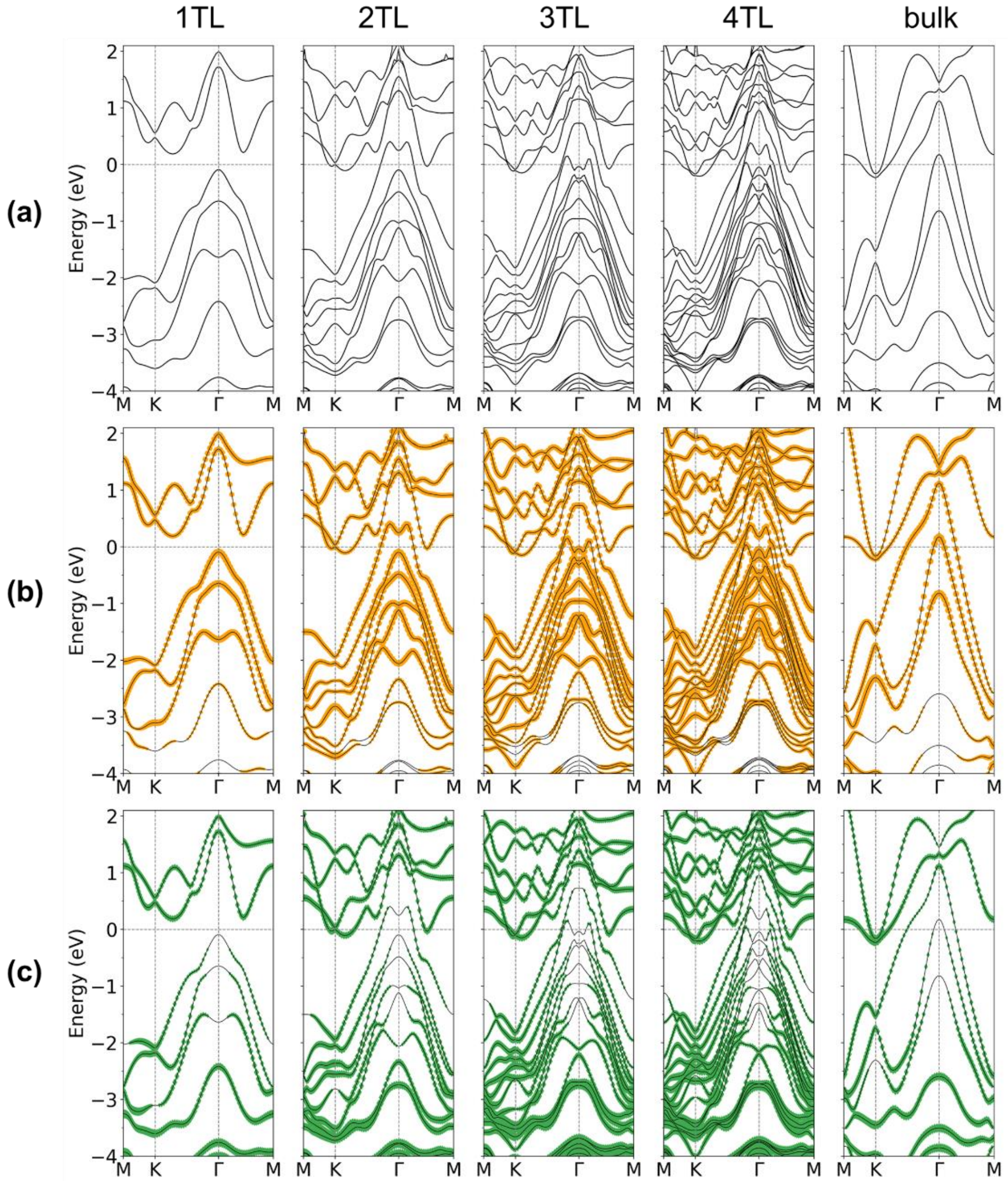

Figure S6. Calculated band structure of primitive $PtTe_2$. (a) Total band structure; Projected band structure for (b) *p*-orbital (orange spheres) and (c) *d*-orbital (green diamond), where the size of the symbol indicates the orbital contribution at each *k* point.

With a view to investigating the electronic structure of $PtSe_2/PtTe_2$ heterostructures, we first constructed commensurate supercells in which a $PtSe_2$ (13 × 13) supercell is vertically stacked on a $PtTe_2$ (12 × 12) supercell, corresponding to a lattice mismatch of ~1.2%. For Pt-based TMDs, spin–orbit coupling (SOC) plays a crucial role in shaping the electronic structure. To establish a physically reasonable model applicable for electronic structure calculations, we systematically examined four strain distribution schemes (as illustrated in **Figure S7**): (a) 1.3% compressive strain on the $PtSe_2$ and no strain on $PtTe_2$ (bottom Te atoms fixed); (b) 1.3% tensile strain on the $PtTe_2$ and no strain on $PtSe_2$ (bottom Te atoms fixed); (c) balanced strain distribution with 0.65% compressive and tensile strains on $PtSe_2$ and $PtTe_2$, respectively (bottom Te atoms fixed); (d) same as (c), but with the bottom Se atoms fixed.

Total energy comparisons indicate that configurations (b) and (c) are both energetically preferred, with only ~60 meV energy difference. However, configuration (c) distributes strain more evenly between the two layers, thereby minimizing excessive lateral deformation for both layer and better preserving the intrinsic structural and electronic characteristics of both materials. In contrast, configuration (d) is energetically unfavorable. Therefore, the averaged lattice constant model corresponding to configuration (c) was adopted for subsequent analysis.

The unfolded band structure of $PtSe_2$ (13 × 13)/$PtTe_2$ (12 × 12) without SOC is shown in **Figure S8**, where emergent bands appear near the Γ point at ~ –6.8 eV (energy is aligned to vacuum). These emergent energy states are due to the interlayer coupling of $PtSe_2/PtTe_2$, which are also observed in **Figure 2(e)** and evidenced in **Figure 4.**

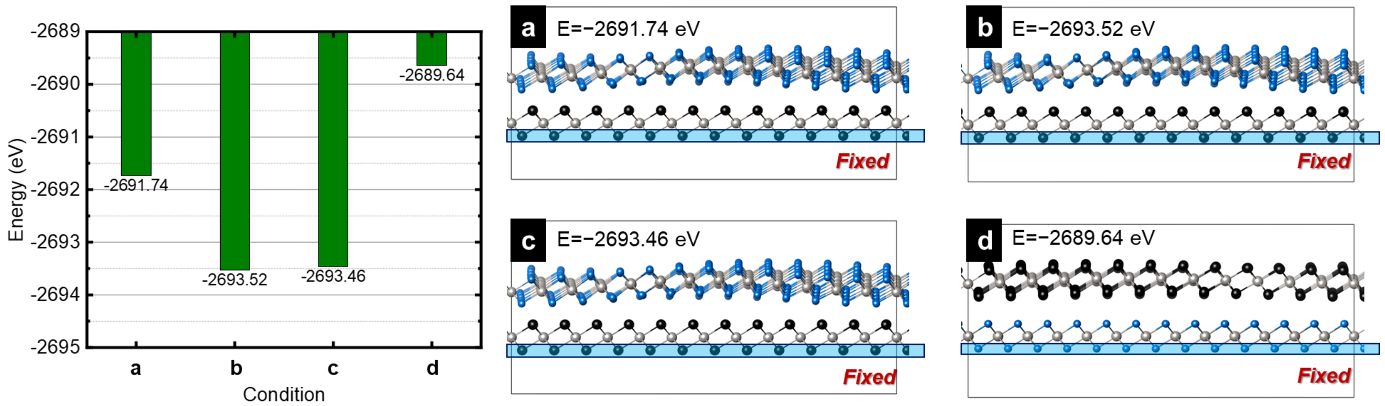

Figure S7. Optimized $PtSe_2/PtTe_2$ heterostructures under four strain conditions: (a) $PtSe_2$ under 1.3% compressive strain and $PtTe_2$ unstrained; (b) $PtSe_2$ unstrained and $PtTe_2$ under 1.3% tensile strain; (c) $PtSe_2$ under 0.65% compressive strain and $PtTe_2$ under 0.65% tensile strain, with bottom Te atoms fixed; (d) $PtSe_2$ under 0.65% compressive strain and $PtTe_2$ under 0.65% tensile strain, with bottom Se atoms fixed. The corresponding energy diagram indicates that conditions (b) and (c) are energetically more favorable than the other two cases. The blue, black, and grey balls are Se, Te, and Pt atoms, respectively.

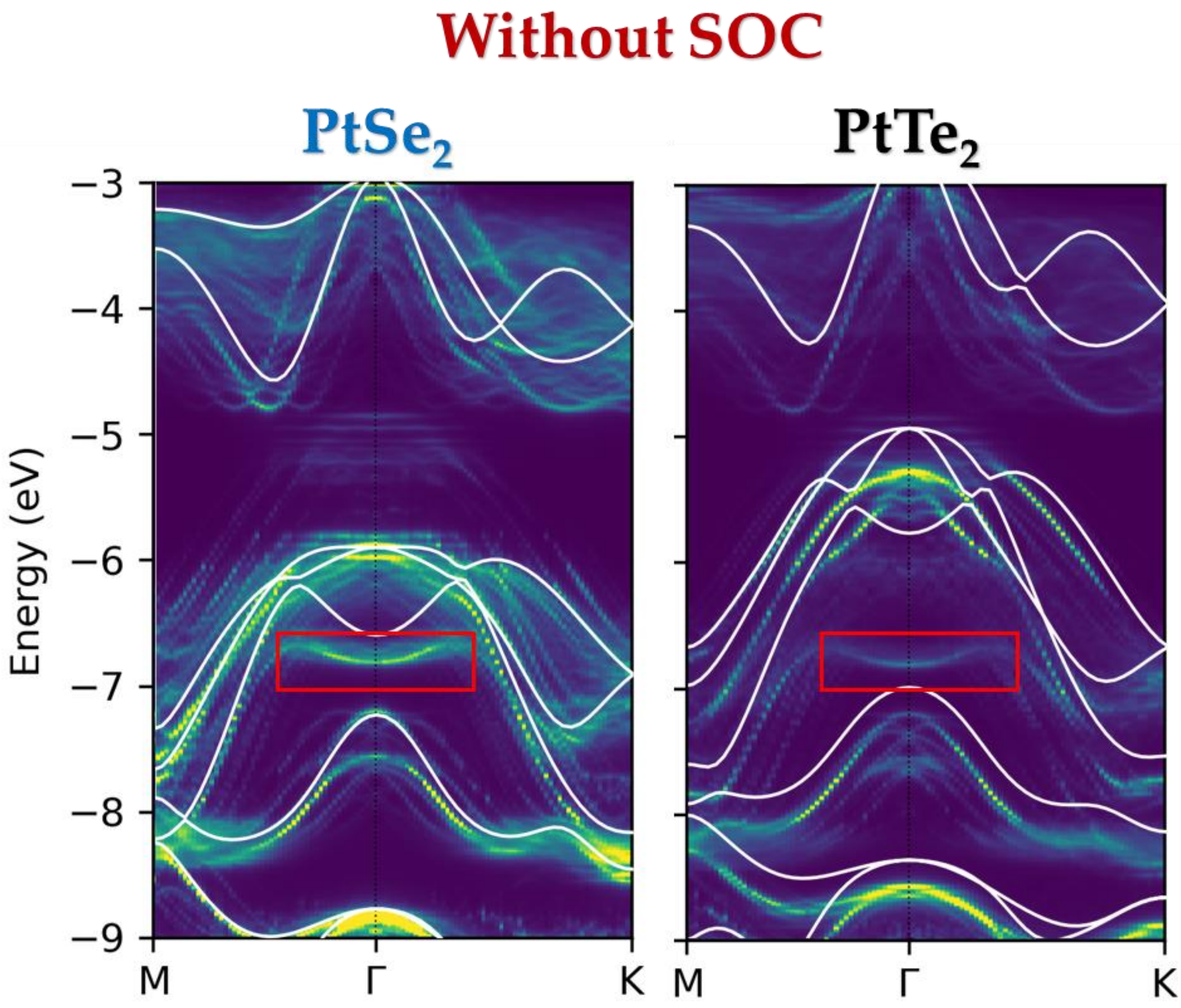

Figure S8. The unfolded band structure for 1TL-$PtSe_2$/1TL-$PtTe_2$ without SOC effect. The energy is aligned to vacuum level and band structure overlaid with the corresponding pristine band structure without SOC effect.

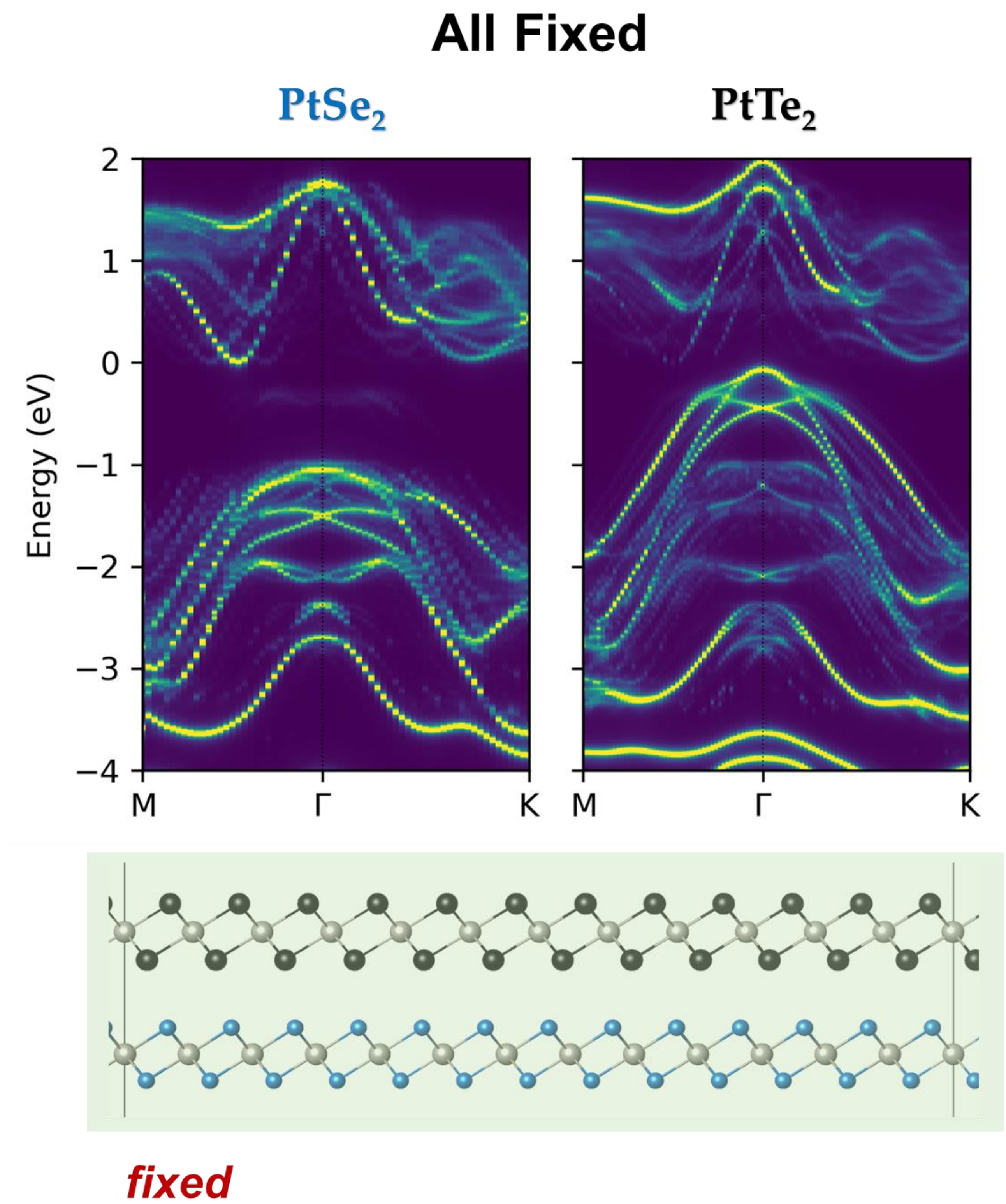

Figure S9. The unfolding band structure of 1TL-$PtSe_2$/1TL-$PtTe_2$ heterostructure model under two conditions: (a) The interlayer distance between $PtSe_2$ and $PtTe_2$ is set to be ~14 Å to simulate the unattached condition; (b) The interlayer distance between $PtSe_2$ and $PtTe_2$ is set to be ~3.4 Å to simulate the formation of 1TL-$PtSe_2$/1TL-$PtTe_2$ heterostructure.

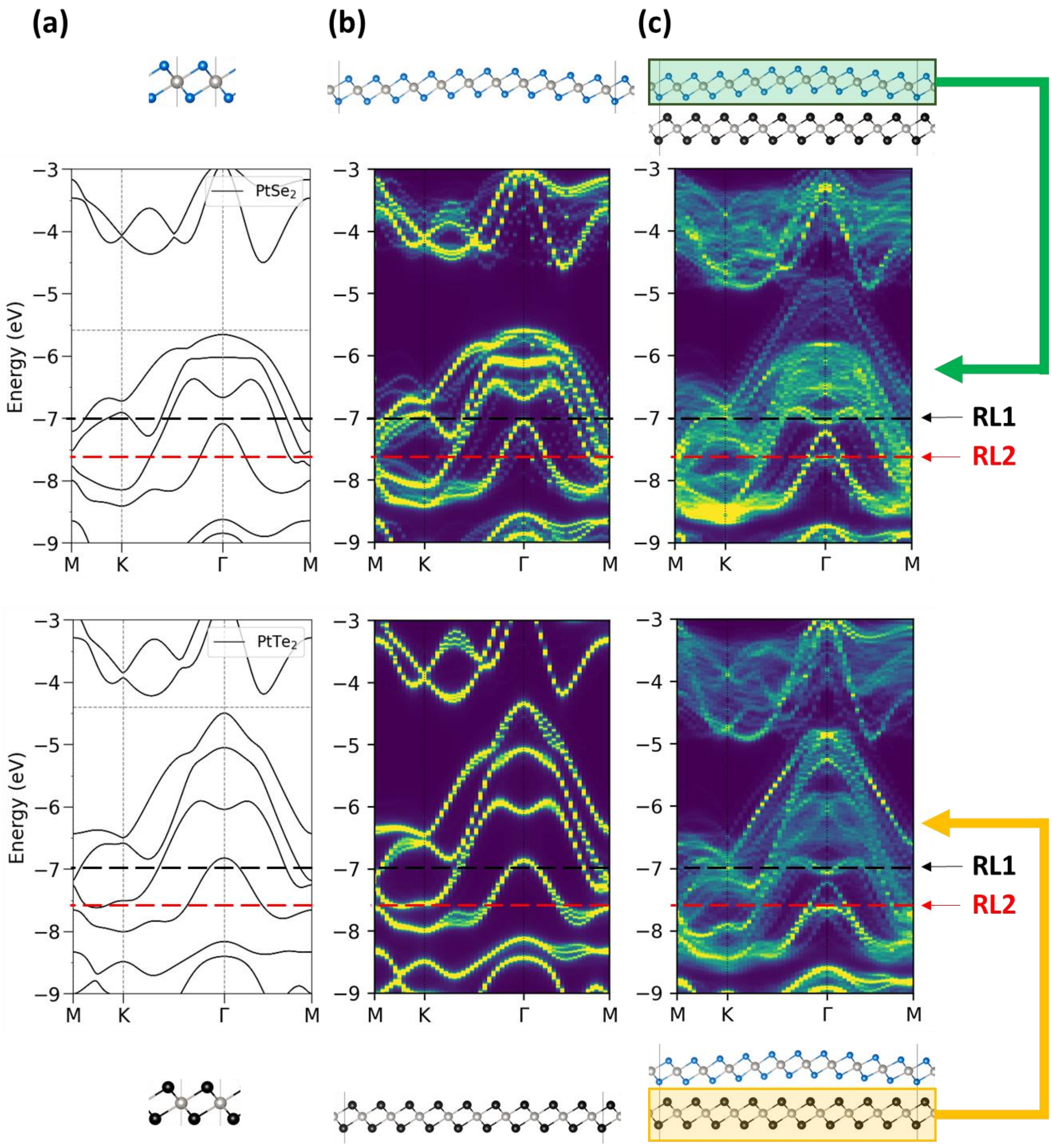

Figure S10. Band structure of (a) Pristine $PtSe_2$ and $PtTe_2$. (b) freestanding 1TL-$PtSe_2$ and 1TL-$PtTe_2$, where the structure is directly decoupled from 1TL-$PtSe_2$ ($\sqrt{84} \times 1$)/1TL-$PtTe_2$ ($\sqrt{73} \times 1$) heterostructure model. (c) The band structure of 1TL-$PtSe_2$ ($\sqrt{84} \times 1$)/1TL-$PtTe_2$ ($\sqrt{73} \times 1$). Two reference lines (RL) are indicated as RL1 (black dash line) and RL2 (red dash line), respectively. The energy is aligned to vacuum level.

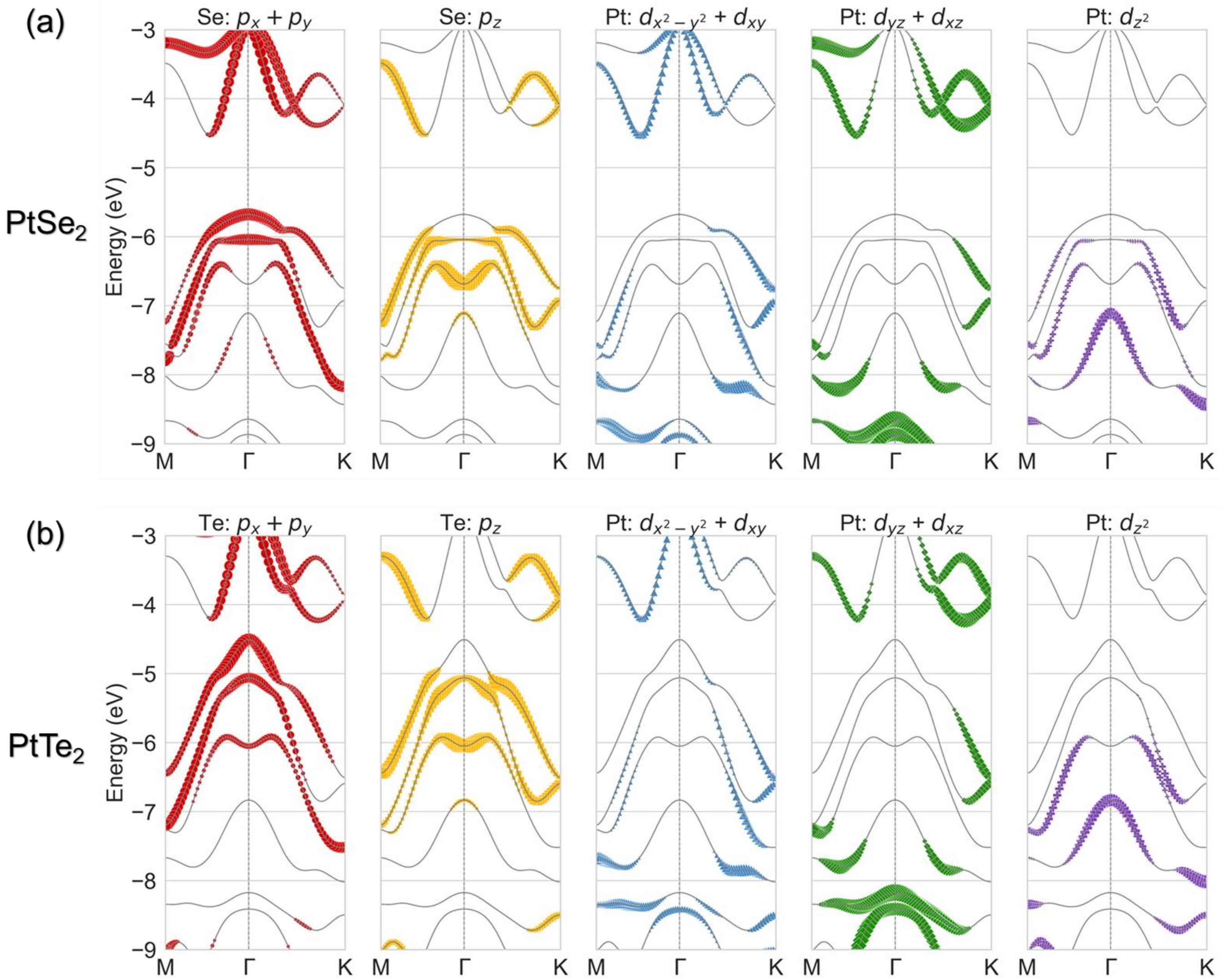

Figure S11. Orbital projected band structure of (a) Pristine 1TL-$PtSe_2$ and (b) Pristine 1TL-$PtTe_2$.

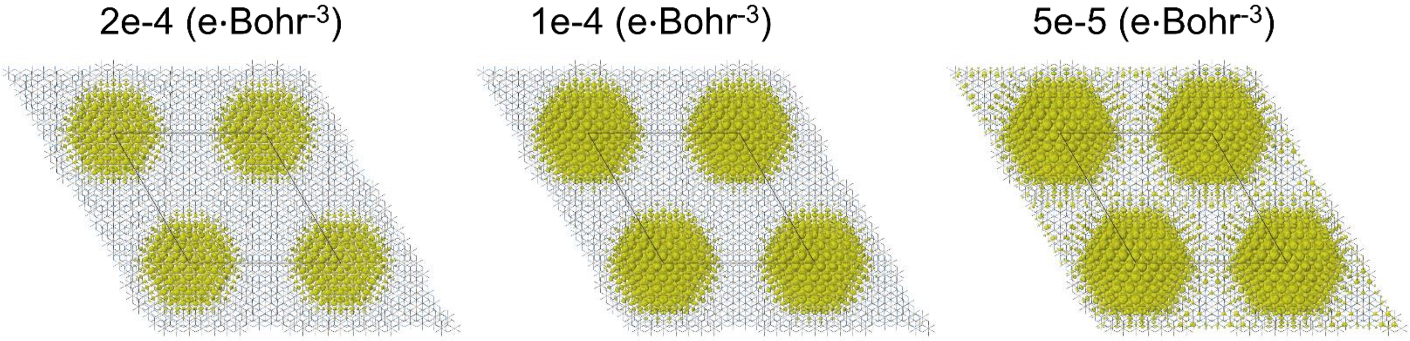

Figure S12. Comparison of partial charge density isosurfaces using the isovalue adopted in Figure 4 of the main text (1×10^{-4} e · $Bohr^{-3}$) together with higher and lower thresholds. All isosurfaces exhibit the same C_3 symmetry and pronounced localization at the MM site, demonstrating that the observed spatial confinement is robust against variations in the chosen isovalue.
